# Patient and Healthcare Provider Experience With Rheumatoid Arthritis in Northern Ontario, Canada: A Qualitative Descriptive Study

**DOI:** 10.1002/msc.70015

**Published:** 2024-11-26

**Authors:** Nancy Lightfoot, David Marsh, Sherry Mongeau, Susan Boyko, Behdin Nowrouzi‐Kia, Lucio Fabris

**Affiliations:** ^1^ School of Kinesiology and Health Sciences Laurentian University Sudbury Ontario Canada; ^2^ Human Sciences Division Northern Ontario School of Medicine University Sudbury Ontario Canada; ^3^ Clinical Sciences Division Northern Ontario School of Medicine University Sudbury Ontario Canada; ^4^ Department of Occupational Science and Occupational Therapy University of Toronto Toronto Ontario Canada; ^5^ Med‐I‐Well Services Sudbury Ontario Canada

**Keywords:** patient experience, provider experience, qualitative, rheumatoid arthritis

## Abstract

**Background:**

Rheumatoid arthritis (RA) is a disabling common chronic inflammatory joint disease. In Ontario, the burden is higher in those aged 65 and older, in females, and in northern communities. This study examined patient disease impact and healthcare provider access and satisfaction as well as provider satisfaction, patient experience and educational suggestions.

**Methods:**

Semi‐structured interviews and reflexive thematic analysis were used.

**Results:**

Interviews occurred with: (1) 18 Northern (N) Ontario patients, (2) 6 N Ontario family physicians, (3) 6 N Ontario pharmacists and (4) a rheumatologist and 4 advanced clinical practitioners in arthritis care (ACPACs) who treat N Ontario patients. Patients emphasised the need to: (1) act on early symptoms, (2) self‐advocate, (3) attract more N Ontario rheumatologists, (4) educate the public, (5) recognise that medication can change over time and (6) pace physical tasks. Satisfaction was expressed with providers. Family physicians mentioned the need to: (1) be front‐line educators, (2) commence initial treatment, (3) enhance undergraduate medical curricula and (4) require rheumatology rotations. Pharmacists expressed: (1) acting as patient educators, (2) assisting with insurance plans, (3) encouraging family physicians to commence treatment, (4) monitoring medication interactions and (5) professional collaboration. The ACPACs and rheumatologist stressed the value of: (1) patient advocates, (2) family physicians initiating treatment, (3) pharmacists monitoring for drug interactions, (4) expanding undergraduate medical school rheumatology curricula and (5) accessing local care.

**Conclusion:**

Additional patient and public education are needed. Enhancing undergraduate and graduate medical school rheumatology curricula, rotations, continuing rheumatology education and interprofessional collaboration were recommended.

## 1 Background

Rheumatoid arthritis (RA) is the most frequent chronic inflammatory joint disease (Littlejohn and Monrad 2018; Widdifield et al. 2015). In 2020, 1.2% or about 374,000 Canadians, aged 16 or more experienced diagnosed RA, with females (Statistics Canada 2020) and northern, rural and remote communities bearing higher burdens (Liu et al. 2021; Widdifield et al. 2013). Long‐term autoimmune disease bilaterally affects multiple joints, can have physical, occupational, quality of life, mental and social effects and can increase morbidity and mortality (Aletaha and Smolen 2018; Conley et al. 2023; Metsios, Stavropoulos‐Kalinoglou, and Kitas 2015; Parisi et al. 2019). Progressive joint deterioration is associated with pain and stiffness (Sanchez‐Florez et al. 2021). Earlier recognition, rheumatologist referral and initiation of treatment can result in improved long‐term outcomes (Littlejohn and Monrad 2018).

Access to Canadian rheumatologists usually requires family physician referral (Widdifield et al. 2014). Geographic variation in the timely receipt of initial rheumatology care occurs in Canada, with poorer access in rural and remote communities (Widdifield et al. 2014). The number of Canadian rheumatologists remains inadequate (Widdifield et al. 2021).

Anti‐inflammatories such as nonsteroidal anti‐inflammatory drugs (NSAIDs) and glucocorticoids are adjuncts in the early management of pain or inflammation with potential side effects (Littlejohn and Monrad 2018). Pharmacological management of RA has expanded from conventional synthetic disease‐modifying anti‐rheumatic drugs (DMARDS, e.g., methotrexate as first choice, leflunomide, hydroxychloroquine and sulfasalazine) to biological DMARDS and recently to targeted synthetic DMARDS (Conley et al. 2023). A significant number of patients continue to experience pain despite optimal treatment (Sanchez‐Florez et al. 2021).

Both patients and healthcare providers believe that people without RA do not have a good understanding of the associated physical and emotional impact of the disease (Alten et al. 2019). RA has a negative impact on physical, mental and emotional well‐being (Papadimitropoulos et al. 2022), patients favour collaborative care (Hall et al. 2018), and prefer an active role in treatment decisions (Bywall et al. 2022). Pain, fatigue and physical function remain barriers for patients to experience a normal life and patient's relationships, career progression, daily activities and the ability to work can be affected (Alten et al. 2019).

The objectives of this qualitative descriptive study were to determine the following for Northern (N) Ontario RA patients: (1) impact of RA diagnosis, (2) medication experience, (3) experience with access to family physicians and rheumatologists, (4) satisfaction with family physician, pharmacist, and rheumatologist care and (5) family physician, rheumatologist, and advanced clinical practitioners in arthritis care (ACPACs) experience with patients and educational suggestions.

## 2 Methods

### 2.1 Study Design

This study employed a qualitative descriptive design to provide a broad insight where little is known about capturing patient and healthcare provider experiences and perceptions about dealing with RA in Northern Ontario (N Ontario) (Doyle et al. 2020). The design, often used in nursing and healthcare research, does not require a deeply theoretical context and is used to provide straightforward descriptions that stay close to experiences and perceptions while recognising the subjective nature of the problem, especially when little is known (Doyle et al. 2020).

### 2.2 Study Setting, Recruitment and Sampling

Eighteen adult RA patients aged 18 and over and 16 healthcare providers who treat RA in Northeastern (NEO) and Northwestern Ontario (NWO) served areas for rheumatology conditions as well as one provider in Southern Ontario who treats N Ontario patients were recruited. A systematic review found that saturation in qualitative studies was achieved between 9 and 17 interviews, using homogenous and heterogenous populations, with an average of 12–13 interviews (Hennik and Kaiser 2022). While we did achieve saturation in the study, we acknowledge that sample size was also determined based on available funding and the challenge of recruiting busy healthcare providers.

Recruitment occurred between June and September 2022. In this study, rural included those patients living, or providers practicing, in a location with a population under 10,000 and urban with a population of 10,000 and over (du Plessis et al. 2001; Letang 2024).

N Ontario patients were recruited through the Arthritis Society Canada website, RA Canada Facebook site, N Ontario radio and snowball sampling. N Ontario family physicians, rheumatologists, pharmacists and ACPACs (e.g., physiotherapists, occupational therapists, chiropractors and nurses; ACPAC Program Continuing Professional Development 2024) were recruited through the Northern Ontario School of Medicine University (NOSMU) newsletter and emails, the Arthritis Society Canada website, and by snowball sampling. Recruitment of Southern Ontario (SO) rheumatologists who treat some N Ontario patients was attempted using the Arthritis Society Canada website and emails from one author who conducted research with them. N Ontario pharmacists were also recruited using emails and word of mouth from the study pharmacist.

Thus, 35 participants (18 RA patients and 17 healthcare providers) were interviewed with the goal of representing both NEO and NWO. Each study participant received $110.

### 2.3 Data Collection

An interview guide was developed for RA patients and healthcare providers by our interdisciplinary team with epidemiological, rural and northern health, educational, medical (with expertise in pain), occupational therapy and pharmacist expertise. The lead author has RA and travels to a S Ontario rheumatology clinic.

Using an interview guide, semi‐structured interviews were conducted by phone between June and October 2022 by the study research assistant, who also audio‐recorded and transcribed the interviews verbatim.

The interview guide for patients appears in Figure S1. The interview guide for healthcare providers appears in Figure S2.

### 2.4 Data Analysis

The research assistant recorded and transcribed the interviews verbatim. Transcripts were reviewed for accuracy. Interviews were analysed inductively, using manual coding, following Braun and Clarke's reflexive thematic analysis steps: (1) familiarisation with the data by reading and re‐reading, (2) coding to generate labels, (3) generating initial themes by identifying significant broader patterns, (4) reviewing themes by checking against the data, (5) defining and naming themes based on detailed analysis and (6) writing up (Bruan and Clarke 2006, 2019, 2022). Three team members undertook thematic analysis and jointly reviewed the final themes. Differences in coding or interpretation were resolved by discussion. Participants were emailed a summary of the study results.

## 3 Results

The study included interview results from: (1) 12 NEO and six NWO patients, (2) three NEO and three NWO family physicians, (3) five NEO and one NWO pharmacist, (4) two NEO, one NWO, one SO ACPAC and 1 N Ontario rheumatologist. Patient characteristics by participant appear in Table 1, and provider characteristics by participant appear in Table 2 but are limited to retain anonymity and confidentiality. Patients ranging in age from 30 to 76 years had an average age of 58 years (s.d. 15.0). The majority of patients were in the 60–79 years age group, female, from NEO, and resided in urban areas. The majority of providers were female and practiced in N Ontario urban communities.

**TABLE 1:** Patient participant characteristics (*n* = 18).

Participant	Sex	Age group	NEO or NWO resident	Urban or rural
P1	F	3	NEO	U
P2	F	3	NEO	R
P3	M	3	NEO	U
P4	F	3	NEO	U
P5	F	3	NEO	U
P6	M	1	NWO	R
P7	F	3	NEO	U
P8	F	2	NEO	U
P9	M	3	NWO	R
P10	F	2	NWO	R
P11	M	3	NWO	R
P12	F	3	NEO	U
P13	F	1	NEO	U
P14	F	2	NEO	U
P15	F	2	NEO	U
P16	M	2	NWO	U
P17	F	3	NWO	U
P18	F	2	NEO	U

*Note:* Patients (P): *n* = 18. Sex (Female (F): *n* = 13; Male (M): *n* = 5). Age groups (1–3, 1 = 18–39, 2 = 40–59, 3 = 60–79 years): 1: *n* = 2, 2: *n* = 6, 3: *n* =10). Northeastern Ontario (NEO): *n* = 12. Northwestern Ontario (NWO): *n* = 6. Urban (U): *n* = 13, Rural (R): *n* = 5.

**TABLE 2:** Healthcare provider characteristics.

Provider	Sex	N or S Ontario practice	Urban or rural
FP1	F	N	U
FP2	F	N	U
FP3	M	N	R
FP4	F	N	U
FP5	F	N	U
FP6	F	N	U
Ph1	M	N	U
Ph2	F	N	U
Ph3	F	N	U
Ph4	M	N	U
Ph5	F	N	U
Ph6	F	N	U
R1	M	N	U
ACPAC1	F	N	U
ACPAC2	F	N	U
ACPAC3	F	S	U
ACPAC4	F	N	U

*Note:* Family physician (FP): *n* = 6. Pharmacist (Ph): *n* = 6. Rheumatologist (R): *n* = 1. Advanced Clinical Practitioners in Arthritis Care (ACPAC): *n* = 4. Sex (Female (F): *n* = 13; (Male (M): *n* = 4). Northern Ontario (N): *n* = 16, Southern Ontario (S): *n* = 1. Urban (U): *n* = 16, Rural (R): *n* = 1.

### 3.1 RA Patient (P) Interviews

Interviews averaged 60 min. The themes that emerged appear below.

#### 3.1.1 Theme 1: Need to Act on Early Symptoms

Patients expressed the importance of recognising their symptoms and getting diagnosed and treated for early RA symptoms to prevent long‐term effects. The importance for patients to access primary care early in the disease process was emphasised. One expressed: ‘An early diagnosis and starting treatment can prevent those long‐lasting impacts’ (P8). Another said: ‘It is so dangerous if you don't get early care; you will only get worse. You need to be diligent and respectful of your own body’ (P17).

#### 3.1.2 Theme 2: The Need to Self‐Advocate

Participants stressed the need to be pushy, fight for answers and seek treatment when dealing with symptoms. Given that the disease and ongoing symptoms may not be well recognized by primary healthcare professionals, the patient must advocate for answers and treatment to improve comfort and quality of life. One responded: ‘You need to be your own healthcare advocate. You need to fight for the answers, if you know something is wrong’ (P13). Another said: ‘You need to be a bit pushy. You need to advocate and be persistent when managing your symptoms’ (P8). Another emphasised: ‘Get the help, get the physicians, get the rheumatologists, get the Arthritis Society on your side. Get as much information as you can about RA; knowledge is power; you need to know your own body’ (P2).

#### 3.1.3 Theme 3: Need to Attract More Northern Ontario Rheumatologists

Many expressed the need for more rheumatologists in N Ontario, long time periods to acquire rheumatology appointments, and the requirement for long‐distance travel for rheumatology care outside N Ontario, with associated cost and time. One said: ‘Well, we definitely need more rheumatologists in N Ontario’ (P12). Another responded: ‘We just need more rheumatologists’ (P14). Another expressed: ‘I feel like it is so hard to see a specialist in N Ontario. I should not have to travel for care’ (P7). Interestingly, one family physician offered this suggestion for attracting more rheumatologists in N Ontario:

We need to make sure that the compensation for living in N Ontario is adequate and if necessary provide a signing bonus or relocation assistance. It is also important to think about their partners and ensuring that they can find job opportunities as well.

(FP6)

#### 3.1.4 Theme 4: Need to Educate the Public

Given that the public and RA family members frequently do not recognise the severity and impact of RA on patients, participants expressed the need for public education. One indicated:

There is not enough education about this disease…I think there should be pamphlets we can give to our caregivers and immediate family members because people don’t understand it. I would like to see a lot more education…A lot of people don’t know that it can affect your organs.

(P5)

More about this was voiced in this way:

I think somebody has to figure out on a global scale to get rid of the word arthritis. Because I have seen that people have looked at me and said, oh, you only have arthritis. It is such a big stigma, and people don't understand RA. I tell people that I have RA disease.

(P17)

#### 3.1.5 Theme 5: Need to Recognise That Medication Journeys Occur

Many patients described frequent medication changes. They emphasised the need to communicate with rheumatologists when medication was not working and when changes were needed. Dealing with RA often involves a medication a journey over time. One said: ‘I started on prednisone. I tried methotrexate. I tried leflunomide. I tried biologics, and I was taking them by injection. Then, a couple of others that gave me diarrhoea, so I had to stop those…I tried so many’ (P17). Another noted:

I started off with methotrexate. The methotrexate did not work but it took a few years to figure that out. Then I started on Arava^TM^ [leflunomide] which I am still on and I tried Humira^TM^ [adalimumab] and plaquenil^TM^ [hydroxychloroquine], but that did not work. Humira^TM^ injections got me somewhat stable. At one point, I got a bad infection and had to stop Humira^TM^. I am now on Xeljanz^TM^ [tofacitinib].

(P2)

#### 3.1.6 Theme 6: Need to Pace Physical Tasks

RA patients recognized their physical limitations and frequent need to break up physical tasks. One emphasised: ‘I know what I can and cannot do. Sometimes you pace yourself. If you are cutting your lawn, you only do half at a time’ (P4). Another voiced: ‘I take a lot of breaks. If I go outside to work in the garden, it kills me. I still do it, but I take a lot of breaks. I rest now more than I ever did’ (P8). And another expressed:

For the most part, I can do the work without hesitation, but I am also weary that I do not go overboard to save my strength. I am adjusting and modifying. I have learned to do what I can to ensure I am not causing any additional pain. The more active I am, the more my joints feel better.

(P16)

### 3.2 Family Physician (FP) Interviews

Interviews averaged 30 min. The themes that emerged appear below.

#### 3.2.1 Theme 1: Need to Be Front‐Line Patient Educators

As it can take a while to obtain initial rheumatology appointments, participants indicated that the onus is often on the family physician to perform tests to enable initial diagnosis and treatment. One said: ‘So, I often become the front‐line educator because of the wait time to see a rheumatologist’ (FP4). Another added: ‘So often I act as the educator with patients because the patient is not going to be seeing the specialist immediately’ (FP5). Although another said: ‘I find that most of the patients who have RA are educated about their diagnosis and sometimes educate me’ (FP3).

#### 3.2.2 Theme 2: Need to Commence Initial Treatment?

While the family physician can provide initial medications such as prednisone and options for pain management, the commencement of first‐line RA treatment with methotrexate is usually the initial choice, but not all family physicians were comfortable initiating that. One said: ‘For those who aren't diagnosed, it is difficult for me trying to initiate treatment, so I am comfortable prescribing methotrexate if I know that their labs and x‐rays point to RA’ (FP3). Another responded: ‘Most family physicians are not comfortable prescribing front‐line medications for RA patients. I would never prescribe methotrexate without checking with the rheumatologist first’ (FP2).

#### 3.2.3 Theme 3: Need to Enhance Medical Curricula, Particularly About Biologics

Family physicians stressed that more pharmacology education is required at the undergraduate and family physician training levels, especially for biologics. They recommended greater collaboration with pharmacists regarding pharmacologic options. As one expressed:

There is a fear of prescribing medications that they don’t understand. They need more pharmacology, especially for biologics…There has to be more training for the family physicians on medications, especially in rural and remote areas, so they can maintain the patient treatment.

(FP1)

And another also indicated: ‘I think they need to really focus more on pharmacology’ (FP2). Another voiced:

I think it would be good if they could provide us with more education about how to treat an RA patient and how to dispense the difficult medications. An educational refresher would be great so that we have a good idea of what symptoms to look for in RA patients.

(FP3)

#### 3.2.4 Theme 4: Need to Require Rheumatology Rotations

Family physicians expressed the need for rheumatology rotations at both undergraduate and residency levels and more musculoskeletal education about RA and RA clinical experience. One participant said:

I think during the 4th year UME students should have early exposure to rheumatology, which would help them to be able to identify symptoms of RA. Also, students could do research with rheumatologists that might help them build an interest in rheumatology as a specialty.

(FP3)

Another expressed: ‘I think in family physician residency, having a mandatory rotation with rheumatology because rheumatology is such a black box and such a difficult specialty’ (FP3). Another said, ‘It is really important to learn the musculoskeletal exam for RA’ (FP4).

Continuing medical education to support earlier identification of RA was also recommended, such as ‘Having more conferences about RA would be great. We are trying to revamp the CME (continuing medical education) type of event now. It is important to keep family physicians up to date, especially with the new medications like the biologics’ (FP4). Similarly, another expressed: ‘So having some CME‐type events to support family physicians. It is important to establish a relationship between the family medicine physician and the specialist’ (FP6).

#### 3.2.5 Theme 5: Need to Practice Collaborative Care

Some family physicians indicated the need for, and advantages of, collaborative care for RA patients. One family physician voiced: ‘I think there needs to be more emphasis on collaboration with other healthcare professionals like occupational therapists. This will help in having a team‐based approach to the care of the patient’ (FP6).

Another family physician said:

I collaborate a lot with the pharmacist. So I just call the pharmacist and try to collaborate about the medications that they are taking so that there is no confusion for the patient. The pharmacist and I try to ensure we are controlling pain without the patient becoming addicted. It is really important to use appropriate pain management for any RA patient…it is a disease that requires a lot of trial and error with medications.

(FP1)

### 3.3 Pharmacist (Ph) Interviews

Interviews averaged 30 min. Themes that emerged appear below.

#### 3.3.1 Theme 1: Acting as Patient Educators

Access to pharmacists can be easier than accessing family physicians and rheumatologists, and pharmacists were willing to fill gaps and find solutions about proper use, storing, taking, and renewing medication, and checking for potential medication interactions and side effects. One indicated:

Something like RA can be an overwhelming diagnosis for a lot of people. The rheumatologist does a great job of setting the stage and then when the patient comes to us, we can help them with their experiences. I feel like we can help fill in the gaps for the patient. We are easier to access than the rheumatologist.

(Ph5)

That participant also expressed: ‘There is a lot of counselling that goes on with RA patients than with other types of patients’ (Ph5). Another added: ‘It is really important to use the pharmacy as an education tool because we can contact the doctor if it is needed. We can also provide education for injection training’ (Ph4).

#### 3.3.2 Theme 2: Assisting With Insurance Plans

Navigating the systems of insurance companies and forms to obtain medication approval can be time‐consuming and frustrating. Pharmacists were ready to assist patients. One said:

Medications need to be affordable or covered by a drug plan. So having more resources to help the patient navigate through that I feel would be beneficial. It can be very confusing for the patient to get phone calls from a drug company or fill out forms. I help the patient to educate them on the drugs and on the system and on their drug plans.

(Ph3)

#### 3.3.3 Theme 3: Encouraging Family Physicians to Commence Treatment

Pharmacists stressed the importance of early treatment by family physicians and offered to encourage family physicians to initiate treatment. Although not all family physicians were comfortable initiating treatment, pharmacists stressed that they could assist in making this possible. One said, ‘It is very important that you get started on treatment right away. It would be good if family doctors start treatment right away and let us know that they are an RA patient’ (Ph6). Another added: ‘So really, they need to have a better understanding of RA medications. Because the family physician is apprehensive to prescribe methotrexate, the patient suffers because they are not given an appropriate medication first to treat the RA’ (Ph4). Another indicated: ‘It is important for them to understand the patient and have a baseline appreciation and knowledge for the challenges a patient with RA faces. They need to grow empathy and compassion for the patient’ (Ph3).

#### 3.3.4 Theme 4: Monitoring Medication Interactions

Pharmacists indicated their roles in monitoring for medication interactions given their knowledge of full patient medication histories. Pharmacists indicated their roles in monitoring for medication interactions given knowledge of full patient medication histories. One pharmacist stressed the importance of ‘making sure there are no drug interactions’ (Ph2). Another explained their ability to provide advice on side effects, indicating: ‘the side effects can be very hard on patients’ (Ph6).

#### 3.3.5 Theme 5: Advocating for Professional Collaboration

Pharmacists stressed the benefit and desire to be considered part of the patient care team. More interaction with other health care professionals was recommended. One emphasised: ‘The team approach would be the best approach’ (Ph1). Another expressed: ‘We need to be a part of the circle of care…I hope that with medical schools, there is more collaboration with other health professionals’ (Ph6). Yet another noted: ‘There has always been a disconnect between the doctor and the pharmacy…It would be nice to have the direct involvement of a pharmacist team member instead of being on the sideline and trying to put the pieces together’ (Ph5).

### 3.4 Rheumatologist (R) and Advanced Clinical Practitioners in Arthritis Care (ACPAC) Interviews

Interviews averaged 45 min. Despite extensive efforts to include more N Ontario and S Ontario rheumatologists in the study who treated N Ontario patients, only 1 N Ontario rheumatologist was recruited. The themes that arose appear below.

#### 3.4.1 Theme 1: Value of Patient Advocates

These study participants emphasised that patients should educate themselves about disease management, patient resources, risks and benefits of various medications, and patient support programs. The need to actively participate in their own care and communicate with health care professionals was clear. One ACPAC stressed: ‘being engaged in their own care is really important’ (ACPAC3). Another expressed:

I think for the patient probably you need to be your own best advocate. There is nobody who is going to know your body better than you. They really need to be an active player and team member in your care.

(ACPAC1)

Another ACPAC said: ‘Education is so important. They really need to understand how to self‐manage because of their chronic disease. They need to ensure they are aware of programs that are available to them’ (ACPAC4).

Another elaborated:

Really it is about education and advocating for themselves. I think empowering people to make the right decisions for themselves. It is important to connect with the right resources. When they have knowledge and resources, they do so much better overall. Helping them to understand the risks and benefits of medications is really important so that the patient knows they are getting the right care.

(ACPAC2)

#### 3.4.2 Theme 2: Value of Family Physicians Initiating Treatment

The value of family physicians initiating bloodwork and methotrexate treatment while waiting to see the rheumatologist was raised, and one ACPAC emphasised that: ‘many of the rheumatologists are running six to nine‐month waitlists and that is too long because by the time the patient is seen they will already have erosions in their joints’ (ACPAC1). And another stressed:

So in providing education for primary care and getting them comfortable with recognizing and getting the basic blood work and starting the patient on methotrexate. So it is about recognizing, understanding, and starting the appropriate treatment, while they are waiting to see the rheumatologist.

(APAC2)

The rheumatologist also added:

Expand or increase the teaching of rheumatology, so the basics so family physicians can start treatment with the proper drugs such as oral methotrexate…So, this requires further discussion with family physicians so that we can limit the damage that RA can cause.

(R1)

#### 3.4.3 Theme 3: Value of Pharmacists Monitoring for Drug Interactions

Patients may use a variety of medications and supplements, so the pharmacist is well‐placed to monitor interactions. The rheumatologist emphasised: ‘It is important to remind patients to be careful about interactions, so the pharmacists really need to understand what the interactions are’ (R1). An ACPAC indicated that the pharmacist is helpful because: ‘Sometimes what arises is when patients have questions about interactions about other medications they may be on’ (APCAC3).

#### 3.4.4 Theme 4: Value of Expanding Medical School Rheumatology Curricula

Undergraduate medical curricula were described as having minimal rheumatology exposure. Participants emphasised the need to improve rheumatology‐related physical examination and musculoskeletal (MSK) examination, expand the introduction to rheumatology, and be aware patients can have a medical emergency. One ACPAC said: ‘There is not enough information on physical examination for arthritis…it can take years to determine what is swollen and what is not (APAC1)’. Another noted: ‘In medical school they get very little MSK health and the rheumatology component is minimal’ (ACPAC2). The rheumatologist indicated: ‘Expand the teaching of the introduction to rheumatology’ and added: ‘It is important for physicians to know that patients with RA can have a medical emergency’ (R1).

#### 3.4.5 Theme 5: Value of Accessing Local Care

These participants stressed the value of expeditiously accessing local care, reducing wait times and avoiding travel. The need for more rheumatologists in the north was stressed. One ACPAC expressed: ‘We don't have enough rheumatologists in the north’ (ACPAC1). Another ACPAC noted:

It would be ideal to have rheumatologic care consistent in the North so that wait times are shorter, and they do not need to be seen in a larger urban centre that is far from home. If people could access care more locally, they could be seen more quickly, especially if they are in a flare‐up.

(ACPAC3)

ACPACs also indicated that they can assist with triage for initial patients and some followup examinations. One noted: ‘I really try to get to the closest community to my patient’, but was frustrated because: ‘I can't sign a travel grant or order bloodwork or x‐rays which is a nuisance’ and ‘we are not well paid, nor always available in some Northern Ontario communities’ (ACPAC1). Another responded: ‘I was able to go out to the remote communities to provide care and followup (ACPAC4)’.

## 4 Discussion

Patients recommended being proactive, advocating and acting upon early symptoms, recognising that medication journeys can occur, and the benefit of pacing physical tasks. Some family physicians supported commencing initial RA treatment, but others were reluctant. Pharmacists and ACPACs were keen to encourage family physicians to commence treatment.

Lengthy wait times to access rheumatologists and the importance of local care were raised by all types of study participants. Fullerton et al. (2020) described satisfaction when occupational or physical therapists triaged access to some Ontario rheumatologists. Supportive education for patients was also emphasised by all types of study participants. RA patients also expressed the need for more public education.

Suggestions to improve the undergraduate medical curricula came from family physicians, ACPACs, and the rheumatologist. Family physicians recommended enhancement of the undergraduate and family medicine rheumatology curricula. The rheumatologist stressed the value of an enhanced introduction to rheumatology and required rheumatology rotations in the undergraduate and family medical curricula. ACPACs stressed the importance of improved musculoskeletal examinations. A world forum highlighted the need for medical graduates to achieve a basic level of rheumatic and musculoskeletal diseases and initiate appropriate treatment (Al Maini et al. 2020). Patients and ACPACs emphasised the need to attract more N Ontario rheumatologists.

Our results are consistent with the literature, including the need for RA patients to self‐advocate (Wada 2019), act on early symptoms (Barber et al. 2021), physically pace themselves (Ostlund et al. 2016) and for more rheumatologists, especially in northern and rural communities (Widdifield et al. 2014), medication journey experiences (Wada 2019) and more public education (Simons et al. 2017). Pharmacists indicated that they could assist patients with insurance documentation. Consistent with the literature, pharmacists emphasised their educator role (Simons et al. 2022) and advocated for more interprofessional team‐based collaboration (Marion and Balfe 2011). The rheumatologist and ACPACs advocated for more undergraduate musculoskeletal and rheumatology medical education (Harkins, Burke, and Conway 2023), the value of local care and additional local rheumatologists, and other sources mentioned enhanced training of primary care physicians to diagnose and care for patients in under‐resourced areas (Lennep, Crout, and Majithia 2020).

A review about the application of telemedicine in the management of RA stressed its benefits and recommended more studies to identify the most effective uses and to evaluate alternative health care services for patients with barriers to telemedicine access (Barlas et al. 2023). However, the absence of face‐to‐face contact with health professionals was reported negatively by some and older age, lack of or limited internet access, limited health and digital literacy and satisfaction with telemedicine were potential challenges.

More rheumatologists must be attracted to northern communities in Canada and recommendations are similar for the attraction of various specialists including: training more specialists in northern medical schools, increasing the number of residencies, and ensuring there are other healthcare providers for support (Kleiner 2021).

### 4.1 Strengths and Limitations

Few studies consider both patient and provider experiences with RA (Prothero et al. 2019; Walker and Robinson 2023). This appears to be the first qualitative study of RA experience described by N Ontario patients and N Ontario healthcare providers. Furthermore, it is important to consider options to make the diagnosis and initiate treatment more quickly to limit disease impact for these patients, such as: enhancing the N Ontario undergraduate family physician education about RA diagnosis and treatment, utilising ACPACs more broadly in N Ontario, and recruiting and retaining more rheumatologists to N Ontario. There is a definite need for more interprofessional collaboration using a team‐based approach between N Ontario healthcare providers who treat N Ontario RA patients. The small sample sizes, use of social media and website recruitment, and geographical location may have led to selection bias and limited transferability, yet several findings are consistent with other literature. Not all family physician participants will have trained in N Ontario. As participation by rheumatologists was poor, it may be helpful to determine if emailed questions and including a rheumatologist(s) on the research team could assist in future.

## 5 Conclusions

Patients should self‐advocate, act on early symptoms, recognise that medication journeys occur, and endorse a better public understanding of RA. Enhancement of the N Ontario undergraduate medical and family medicine rheumatology and musculoskeletal education and interprofessional rheumatology‐related practice opportunities are needed. Ways to attract more students to rheumatology practice in N Ontario are required. Greater N Ontario interprofessional collaboration is needed.

## Author Contributions

Nancy Lightfoot designed the study, analysed and interpreted the results, drafted this paper, will approve the final version, and address questions. David Marsh contributed to the design of the study and interpretation of results, contributed to drafting this paper, will approve the final version and address questions. Sherry Mongeau contributed to study design, acquired and interviewed participants, contributed to data analysis and interpretation, contributed to drafting this paper, will approve the final version and address questions. Susan Boyko participated in study design, analysis and interpretation of results, contributed to drafting this paper, will approve the final version, and address questions. Behdin Nowrouzi‐Kia and Lucio Fabris contributed to study design and interpretation of results, drafting this paper, will approve the final version and address questions.

## Ethics Statement

The Laurentian University Research Ethics Board approved the study (file number: 6020214, April 27, 2022).

## Consent

Written informed consent for semi‐structured telephone interviews was obtained.

## Conflicts of Interest

The authors declare no conflicts of interest.

## Supporting information

Figure S1

Figure S2

## Data Availability

This study involved analysis of qualitative data; therefore, the data generated are not suitable for sharing beyond that contained within the paper.
