# Supplementary material for: Patient and Healthcare Provider Experience With Rheumatoid Arthritis in Northern Ontario, Canada: A Qualitative Descriptive Study
Source: Musculoskeletal Care. 2024 Nov 26;22(4):e70015. doi: 10.1002/msc.70015 (PMC11599164; doi:10.1002/msc.70015)
Supplement: Supplementary file 1 — Figure S1 [file MSC-22-e70015-s001.docx]

**SUPPLEMENTARY FIGURE 1. Patient Interview Guide**

**Patient Inclusion Criteria**

- **Current resident of northern Ontario (Northeastern (NEO) or Northwestern Ontario (NWO))**
- **English speaking**

**Patient Exclusion Criteria**

- **Deaf**

ID #_________________________

Date of Interview (day/month/year): _______________________

NEO, NWO, or Southern Ontario: __________________________

Urban, Rural, or Remote: ________________________________________

**Introduction:**

*Hello, my name is Sherry and I want to thank you again for participating in this interview. The goal is to share your experience with rheumatoid arthritis; there are no right or wrong answers to the questions asked. Feel free to ask me to repeat the question or explain if you do not understand the question. You may also choose not to answer a question. If you need a pause or a break, we can stop the interview at any time. Do you have any questions before we start?*

**Patient Participant Questions:**

1. How long have you lived with RA (rheumatoid arthritis)? When was it diagnosed by a rheumatologist?
2. Tell me a little about your initial symptoms and how your RA was diagnosed.
3. How long did it take to get the first rheumatologist appointment? What type of appointment was it and where? What is the distance from your home to where the rheumatologist practices?
4. Tell me about the impact of RA on you? How has your family reacted to it?
5. What drugs have been prescribed for your RA (start from the beginning and go in order up until now)? How have you handled pain? Have you ever tried low-dose naloxone? Do you take biologics and if so, which ones, and how do you get them? Do you prefer oral or injectable biologics or biosimilars and why? Do you have coverage for your prescribed drugs? Do you use any supplements and if so which ones?
6. If you ever took prednisone, what was your experience like with tapering prednisone?
7. How would you describe your current quality of life? What do you find physically challenging? Does RA affect you emotionally?
8. How satisfied are you with the care/help you have received so far from: your family physician, nurse practitioner, rheumatologist, pharmacist, ophthalmologist, optometrist, chiropractor, physiotherapist, occupational therapist, naturopath, massage therapist, and any others?
9. What do you think doctors and other healthcare professionals should learn more about while they are training to better care for people with RA?
10. How easy is it for you to reach your pharmacist?
11. What was your experience with RA like during the COVID-19 pandemic? Did you get the vaccine? When did you get the vaccine? If so, do you know which one you got? Did you have any side effects?
12. Do you currently work? If so, what are the job title and duties? Do you have any accommodations in the workplace? Does your employer know about the RA? Do coworkers know about the RA?
13. Do you receive any type of compensation for RA? What is it like to deal with compensation organizations?
14. What suggestions do you have for others with RA?
15. Is there anything else you would like to suggest or add?
16. (THIS QUESTION IS ASKED ONLY IF RECRUITMENT IS SLOW): Is there anyone else who lives in NEO/NWO that you could recommend we talk to? If so, would you be willing to provide an email address or telephone number for them?

**Other prompts for all:**

- Please explain more.
- Can you give me more details?

Demographic:

1. How old are you?
2. Where do you live?
3. Do you live with anyone and if so whom?
