# Supplementary material for: Patient and Healthcare Provider Experience With Rheumatoid Arthritis in Northern Ontario, Canada: A Qualitative Descriptive Study
Source: Musculoskeletal Care. 2024 Nov 26;22(4):e70015. doi: 10.1002/msc.70015 (PMC11599164; doi:10.1002/msc.70015)
Supplement: Supplementary file 2 — Figure S2 [file MSC-22-e70015-s002.docx]

**SUPPLEMENTARY FIGURE 2. Healthcare Provider Interview Guide**

**Healthcare Provider Participant Questions**

**Note: Indicate where currently practice and if urban, rural, or remote location**

**Family Physician Questions**

**Inclusion: Family physicians, with patients newly diagnosed with rheumatoid arthritis (RA), by a rheumatologist, who currently practice in Northeastern Ontario (NEO) or Northwestern Ontario (NWO).**

ID # _______________________

Date of Interview (day/month/year):

__________________________

*Hello, my name is Sherry and I want to thank you again for participating in this interview. The goal is to share your stories as you see them; there are no right or wrong answers to the questions asked. Feel free to ask me to repeat the question or explain if you do not understand the question. You may also choose not to answer a question. If you need a pause or a break, we can stop the interview at any time. Do you have any questions before we start?*

1. What is it like to treat patients with rheumatoid arthritis (RA) in northern Ontario?
2. What suggestions do you have for RA patients in northern Ontario?
3. What works well and what needs improvement for RA patients in northern Ontario?
4. How can we attract more rheumatologists to northern Ontario?
5. Do you have some suggestions for rheumatologists who treat northern Ontario RA patients?
6. Is there any information that the rheumatologist could provide to you that would assist you in your care for patients that have rheumatoid arthritis?
7. Do you have any suggestions for ophthalmologists who treat in northern Ontario RA patients?
8. What suggestions do you have for pharmacists who provide care for RA patients in northern Ontario?
9. Do have any recommendations for the undergraduate medical school curriculum?
10. Is there anything else you would like to suggest or add?

11. (THIS QUESTION IS ASKED ONLY IF RECRUITMENT IS SLOW): Are there any other family physicians in NEO/NWO that you could recommend we talk to about their experience with RA patients? If so, would you be willing to provide an email address or telephone number for them?

**Pharmacist Questions**

**Inclusion: Pharmacists who currently practice in Northeastern Ontario (NEO) or Northwestern Ontario (NWO), with rheumatoid arthritis (RA) clients, diagnosed by a rheumatologist.**

ID #__________________________

Date of Interview (day/month/year):

_____________________________

*Hello, my name is Sherry and I want to thank you again for participating in this interview. The goal is to share your experience with patients with rheumatoid arthritis; there are no right or wrong answers to the questions asked. Feel free to ask me to repeat the question or explain if you do not understand the question. You may also choose not to answer a question. If you need a pause or a break, we can stop the interview at any time. Do you have any questions before we start?*

1. What is it like to assist patients with rheumatoid arthritis (RA) in northern Ontario? Do you have any suggestions about how you could further assist patients?
2. What suggestions do you have for family physicians who treat RA northern Ontario patients?
3. What suggestions do you have for rheumatologists who treat northern Ontario patients?
4. Is there any information that the rheumatologist could provide to you that would assist you in your care for patients that have rheumatoid arthritis?
5. Do you have any suggestions for ophthalmologists who treat northern Ontario patients?
6. Do you find yourself acting as the educator?
7. What suggestions do you have for northern Ontario RA patients?
8. Do you have any suggestions about how to improve the medical school curriculum for family physicians and rheumatologists related to RA medication and interaction with pharmacists?
9. Is there anything else you would like to suggest or add?
10. (THIS QUESTION IS ASKED ONLY IF RECRUITMENT IS SLOW): Are there any other pharmacists in NEO/NWO that you could recommend we talk to about their experience with RA patients? If so, would you be willing to provide an email address or telephone number for them?

**Rheumatologist Questions**

**Inclusion: Rheumatologists who currently treat rheumatoid arthritis (RA) patients who reside in Northeastern Ontario (NEO) or Northwestern Ontario (NWO). Aim: 1 NEO and 1 NWO rheumatologist, 2 Southern Ontario rheumatologists.**

ID #___________________________

Date of Interview (day/month/year):

_____________________________

*Hello, my name is Sherry and I want to thank you again for participating in this interview. The goal is to share your experience with patients with rheumatoid arthritis; there are no right or wrong answers to the questions asked. Feel free to ask me to repeat the question or explain if you do not understand the question. You may also choose not to answer a question. If you need a pause or a break, we can stop the interview at any time. Do you have any questions before we start?*

1. What is it like to assist patients with rheumatoid arthritis (RA) in northern Ontario? Do you have any suggestions for improvement?
2. What suggestions do you have for family physicians with RA patients in northern Ontario?
3. Do you have any recommendations for northern Ontario ophthalmologists with northern Ontario RA patients?
4. What suggestions do you have for pharmacists with RA patients in northern Ontario?
5. What suggestions do you have for RA patients in northern Ontario?
6. Do you have any recommendations for improvement in undergraduate and graduate rheumatology curricula?
7. Is there anything else you would like to suggest or add?
8. (THIS QUESTION IS ASKED ONLY IF RECRUITMENT IS SLOW): Are there any other rheumatologists in NEO/NWO/Southern Ontario that you could recommend we talk to about their experience with RA patients? If so, would you be willing to provide an email address or telephone number for them?

**Advanced Clinical Practitioners in Arthritis Care (ACPACs) Questions**

**Inclusion: ACPACs (in Northern and Southern Ontario) who currently treat rheumatoid arthritis (RA) patients who reside in Northeastern Ontario (NEO) or Northwestern Ontario (NWO). Aim: 4 ACPACs.**

ID # __________________________

Date of Interview (day/month/year):

______________________________

*Hello, my name is Sherry and I want to thank you again for participating in this interview. The goal is to share your experience with patients with rheumatoid arthritis; there are no right or wrong answers to the questions asked. Feel free to ask me to repeat the question or explain if you do not understand the question. You may also choose not to answer a question. If you need a pause or a break, we can stop the interview at any time. Do you have any questions before we start?*

1. What is it like to assist patients with rheumatoid arthritis (RA) in northern Ontario? Do you have any suggestions for improvement?
2. What is the role of Advanced Clinical Practitioners in Arthritis Care (ACPACs) in Ontario and specifically northern Ontario?
3. What suggestions do you have for family physicians with RA patients in northern Ontario?
4. Do you have any recommendations for northern Ontario ophthalmologists with northern Ontario RA patients?
5. What suggestions do you have for pharmacists with RA patients in northern Ontario?
6. What suggestions do you have for RA patients in northern Ontario?
7. What did you do your training in to date? Do you have any recommendations for improvement in undergraduate and graduate rheumatology curricula for your discipline?
8. How do we attract more ACPACs in northern Ontario?
9. Is there anything else you would like to suggest or add?
10. Are there any other ACPACs in NEO/NWO/Southern Ontario that you could recommend we talk to about their experience with RA patients? If so, would you be willing to provide an email address or telephone number for them?

**Other prompts for all:**

- Please explain more.
- Can you give me more details?
